# Human Enzymes for Organic Synthesis

**DOI:** 10.1002/anie.201800678

**Published:** 2018-09-11

**Authors:** Margit Winkler, Martina Geier, Steven P. Hanlon, Bernd Nidetzky, Anton Glieder

**Affiliations:** ^1^ Institute for Molecular Biotechnology Graz University of Technology Petersgasse 14 8010 Graz Austria; ^2^ acib GmbH Petersgasse 14 8010 Graz Austria; ^3^ F. Hoffmann-La Roche 4070 Basel Switzerland; ^4^ Institute of Biotechnology and Biochemical Engineering Graz University of Technology Petersgasse 12 8010 Graz Austria

**Keywords:** biocatalysis, biotransformation, drug metabolites, human enzymes, pharmaceutical compounds

## Abstract

Human enzymes have been widely studied in various disciplines. The number of reactions taking place in the human body is vast, and so is the number of potential catalysts for synthesis. Herein, we focus on the application of human enzymes that catalyze chemical reactions in course of the metabolism of drugs and xenobiotics. Some of these reactions have been explored on the preparative scale. The major field of application of human enzymes is currently drug development, where they are applied for the synthesis of drug metabolites.

## 1 Introduction

The value of enzymes as tools for organic synthesis was recognized long ago^1^ and numerous enzyme‐catalyzed reactions have been reported. Today, enzymes are applied widely on industrial scale for example, for the production of fine chemicals, pharmaceuticals, and agrochemicals.^2^, ^3^, ^4^, ^5^ The human being as a source of biocatalysts has mainly been explored in research focused on drug development. In the drug development process, authentic human drug metabolites are required for structure elucidation, as analytical reference, and for toxicology testing. The classical way to identify metabolites of drug candidates is prediction of the expected metabolite structure, often based on LC–MS/MS analysis of samples from incubations with disrupted tissue samples (e.g., human liver homogenate) or microsomal preparations thereof,^6^ and recombinant drug‐metabolizing enzymes contained in bactosomes^7^ or supersomes.^8^ Recently, computational approaches have also gained significance in the prediction process.^9^ Metabolite preparation can become significant in terms of time and cost factors if elaborate multistep chemical syntheses become necessary for their synthesis. This applies especially for chemo‐, regio‐, and stereoselective oxidations, which represent a prominent share of the human body's repertoire of metabolic reactions.^10^ Chemical equivalents to the natural one‐step reaction are not available for the majority of the desired metabolic reactions. One solution to this problem is the use of the human enzymes as catalysts for these transformations.

In human drug metabolism, active pharmaceutical ingredients (APIs) are typically converted into more polar metabolites of their parent compounds to facilitate their excretion.^11^ In this context, enzymatic oxidations play a major role in phase I metabolism, whereas phase II metabolism is dominated by the attachment of glucuronic acid to the phase I metabolites.^6^ However, a significant number of other redox, hydrolytic, or conjugation reactions may also occur,^10^ depending on the chemical structure of the API. The largest number of metabolic reactions is ascribed to the action of cytochrome P450 monooxygenases^12^ (CYPs, phase I) and uridine diphosphoglucuronosyltransferases (UGTs, phase II) but dehydrogenases, hydrolases, glutathione transferases, sulfotransferases, flavin monooxygenases, aldehyde oxidase, xanthine oxidoreductase, and others also act on particular compounds.^6^, ^13^, ^14^ These enzymes can act also in sequence, giving rise to complex product mixtures that strongly depend on the location in which the molecule meets the enzymes and the available enzyme repertoire in the individual of question.

In a few cases, human enzymes have also been applied in the preparation of compounds not related to drug metabolism, for example, steroid derivatives or chiral alcohols, using enzymes such as aldo‐keto reductases and alcohol dehydrogenases. Currently, such cases are rather the exception, although the full potential of human enzymes in this regard has probably not been realized.

This Review is conceived to shed light on chemical reactions that are catalyzed by human enzymes, in particular in the course of the metabolism of drugs and xenobiotics. Whereas some reaction types have been explored on the preparative scale with biocatalysts of human origin, the majority have not. Since the number of reactions taking place in the human body is vast, this Review focuses on reactions that have been carried out in academic and industrial labs on a scale that allows isolation of the products.

## 2 Hydroxylation Reactions

### 2.1 Hydroxylation of Arenes

Oxyfunctionalization, especially of non‐activated C−H bonds, is a major challenge in organic chemistry. Cytochrome P450 enzymes (CYPs) are able to catalyze these reactions. CYPs constitute a superfamily of heme‐containing monooxygenases that insert one atom of molecular oxygen into the substrate molecule in a regio‐ and stereoselective manner. Besides carbon hydroxylation, CYPs are also capable of catalyzing epoxidations, dealkylations, and heteroatom oxygenations, as well as other atypical reactions such as reductions, desaturations, and isomerizations.^15^

In humans, 57 genes coding for CYPs have been identified^16^ and there they play a pivotal role in the metabolism of drugs and xenobiotics and are involved in the biosynthesis of physiologically important compounds such as steroid hormones and vitamins. Human CYPs are predominantly localized in the membrane of the endoplasmatic reticulum with the catalytic domain of the enzyme residing in the cytosol. Besides these microsomal enzymes, mitochondrial CYPs have also been reported that are integral membrane proteins bound to the inner mitochondrial membrane. The membrane‐bound nature of human CYPs makes them difficult expression targets. Thus, appropriate expression systems (i.e., eukaryotic hosts such as yeasts) or smart expression strategies (e.g., truncation of the N‐terminal membrane anchor) are required.

As monooxygenases, CYPs rely on an electron transport system that provides the electrons required for oxygen activation and substrate oxidation. The redox partner of all drug‐ and xenobiotic‐metabolizing CYPs is the NADPH‐cytochrome P450 reductase (CPR), while typical mitochondrial CYPs interact with a two‐component system consisting of a [2Fe‐2S] ferredoxin and an FAD‐containing, NADPH‐dependent ferredoxin reductase.^17^ To reconstitute functional CYP systems and to regenerate the cofactor by exploiting the cell metabolism, reactions catalyzed by CYPs are often performed with whole cells as indicated in the following examples.^18^

**Scheme 1:**
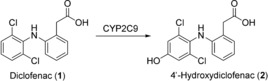
CYP2C9‐catalyzed hydroxylation of diclofenac (**1**).

The non‐steroidal anti‐inflammatory drug (NSAID) diclofenac (**1**) has been used as a substrate for recombinant human CYP2C9 for the preparation of milligram amounts of the 4′‐hydroxy metabolite (**2**; Scheme 1). Such aromatic hydroxylated metabolites can lead to the formation of reactive quinone imines. These electrophiles have been proposed to play a role in mediating the toxicity of NSAIDs through their covalent binding to protein and non‐protein sulfhydryl groups.^19^ Preparation of these metabolites on a milligram to gram scale is therefore desirable to provide material for toxicological investigations. Interestingly, each of the three literature examples employed different expression hosts for recombinant production of CYP2C9. In the first example,^20^ baculovirus‐directed expression in an Sf21 insect cell system was employed for co‐expression of CYP2C9 and CPR. Preparative biotransformations were carried out on a 0.1 L scale and **1** was added to a growing culture at a concentration of 0.25 mm (79 mg L^−1^). The biotransformation proceeded for 55 h and ultimately 2.2 mg of **2** was purified, representing an overall yield of 28 %. In the second example, *E. coli* was employed as expression host.^21^ Biotransformation activities were compared in washed whole cells and isolated cytoplasmic membranes. Although conversion rates and in several cases also final product titers were lower with whole cells compared to membrane preparations, intact cells were chosen for preparative reactions due to ease of biocatalyst preparation. A 1 L reaction was carried out using *E. coli* biomass (100 g L^−1^) and glucose (10 g L^−1^) to support NADPH regeneration. The concentration of **1** was 1 mm (318 mg L^−1^) and the reaction proceeded for 48 h. 110 mg of **2** was purified, representing an overall yield of 35 %. In the third and most impressive example, CYP2C9 was expressed in the fission yeast *Schizosaccharomyces pombe*.^22^ In this case, whole cells (8.3 g L^−1^ dry weight) in phosphate buffer supplemented with 20 g L^−1^ glucose were used for preparative reactions, with 2 mm (636 mg L^−1^) of **1**. After 48 hours, a total of 2.8 g of **2** was purified from six batches, representing an overall yield of 75 %.

**Scheme 2:**
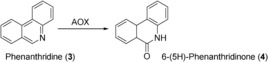
AOX‐catalyzed oxidation of phenanthridine (**3**).

Although the majority of hydroxylations and oxidations described using human enzymes have involved CYPs, other drug‐metabolizing enzymes have also been employed. Aldehyde oxidase (AOX) is a molybdopterin‐containing flavoenzyme that catalyzes nucleophilic attack on electron‐deficient sp^2^‐hybridized carbon atoms, which are typically found adjacent to heterocyclic nitrogen atoms. Molecular oxygen is the terminal electron acceptor in this case.^23^ Human AOX is expressed in the human liver and catalyzes the oxidation of a broad spectrum of aldehydes and aromatic azaheterocycles.^24^, ^25^ This enzyme had proved difficult to produce in large amounts due the absence of the synthetic pathway necessary for the assembly of its molybdopterin cofactor in *E. coli*.^25^ Following extensive investigations into appropriate cultivation conditions, human AOX was, nevertheless, functionally expressed in *E. coli*, as demonstrated by the oxidation of the model substrate phenanthridine (**3**) to 6‐(5*H*)‐phenanthridinone (**4**) on an analytical scale (Scheme 2).^26^

**Scheme 3:**
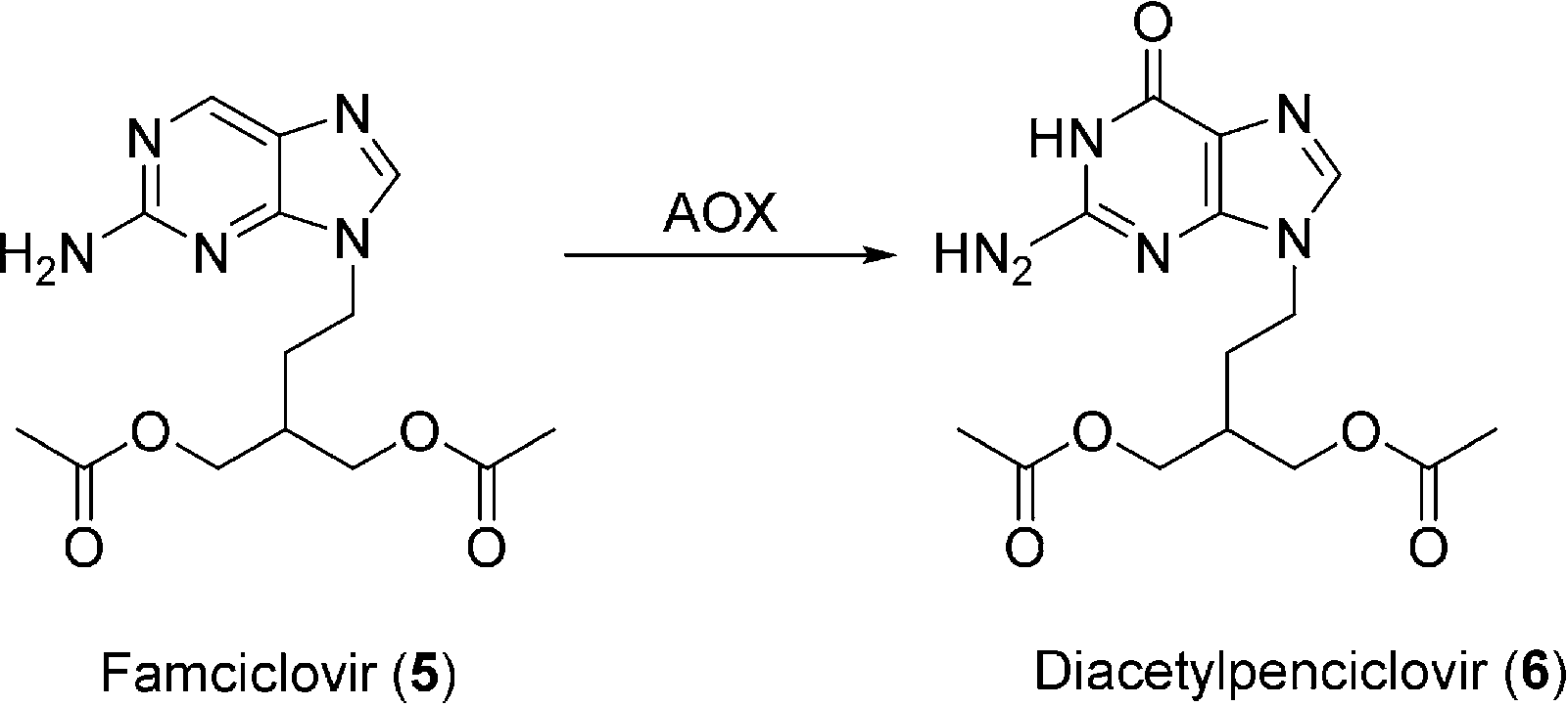
AOX‐catalyzed oxidation of Famciclovir (**5**).

Extensive optimization of biotransformation conditions such as pH, temperature, buffer composition, and solvent addition enabled furthermore the AOX mediated preparation of metabolites of the antiviral drug famciclovir (**5**; Scheme 3). From a 2 L reaction carried out for 3 h at 30 °C in a single‐use bioreactor containing *E. coli* biomass (OD 200) and 210 mg of **5**, 233 mg of the main oxidation product diacetylpenciclovir (**6**) could be isolated as the acetate salt in an overall yield of 82 %.^26^

Like AOX, xanthine oxidoreductase (XOR) is a molybdo‐flavoenzyme, and is characterized by the presence of a molybdenum cofactor (MoCo), a flavin, and two 2Fe/2S redox centers. The overall protein structure and function of the two relatively large and complex enzymes is very similar. The natural and name‐giving substrates of XOR are hypoxanthine and xanthine, however, a wide variety of heterocyclic compounds have been reported as substrates (e.g., purines, pyridines, pyrazine).^24^ Human xanthine oxidoreductase is mostly found in the small intestine and the liver in relatively high amounts,^27^ but also in human milk. XOR exists in two forms, as a dehydrogenase (XDH) and an oxidase (XO), as an effect of post‐translational modification.^28^ XOR was first functionally expressed in an *E. coli* TP1000^29^ *mobAB* deletion strain,^25^ and under well‐optimized expression conditions, the expression also succeeded in a standard *E. coli* BL21 strain. Although the expression levels were extremely low, biotransformation conditions were optimized to reach good conversions within short times, as demonstrated for the oxidation of quinazoline to 4‐quinazolinone. In this case, the optimization of cultivation conditions revealed that highest levels of productivity can be achieved in the absence of inducing agents.^30^ Efficient expression of the human XDH or XOR by other microbial hosts has not been reported so far.

### 2.2 Hydroxylation of Aliphatic Moieties

**Scheme 4:**
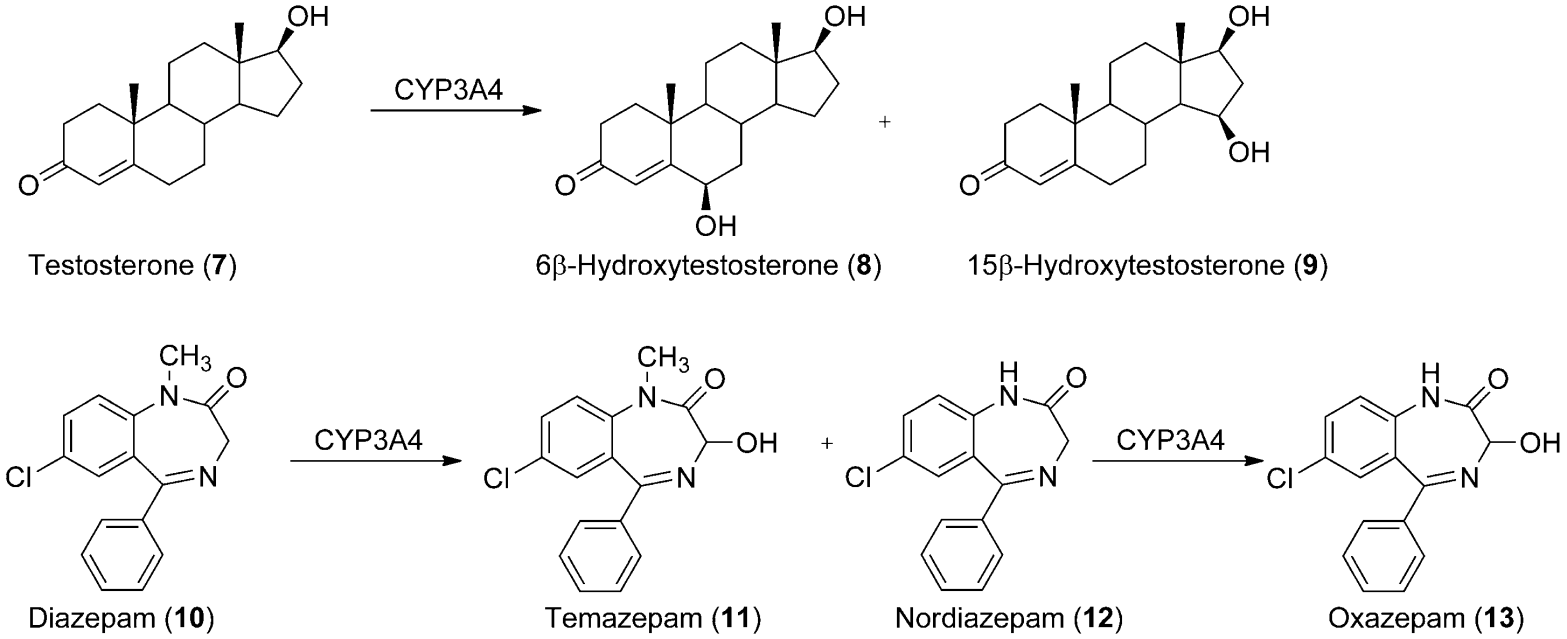
Biooxidation of testosterone (**7**) and diazepam (**10**) catalyzed by human CYP3A4.

Human CYPs also constitute the major enzyme class employed as biocatalysts for aliphatic hydroxylations. CYPs co‐expressed with CPR in Sf21 insect cells were successfully employed to produce, among others, the metabolites resulting from CYP3A4‐catalyzed testosterone (**7**) and diazepam (**10**) hydroxylation (Scheme 4).^20^ The corresponding substrate was added at a final concentration of 100 μm to 0.1 L of a Sf21 suspension culture. After 55 h, conversion of **7** resulted in the formation of 2.3 mg of 6β‐hydroxytestosterone (**8**) and 0.18 mg of 15β‐hydroxytestosterone (**9**) as determined in the reaction supernatant (no product isolation reported). Likewise, **10** was converted into the 3‐hydroxylated product temazepam (**11**, 1.1 mg) by CYP3A4 and to a lesser extent to the N‐demethylated metabolites nordiazepam (**12**, 0.35 mg) and oxazepam (**13**, 0.15 mg).

Although the membrane association of human liver CYPs influences their substrate and product specificity,^31^ and substrate access seems to take place at the membrane/cytoplasm interface, selective 6β‐hydroxylation of **7** on a preparative scale was also demonstrated for *E. coli* expressing an N‐terminally truncated soluble version of CYP3A4 and its reductase^21^ For this purpose, 100 g of washed recombinant cells were resuspended in 1 L of phosphate buffer supplemented with 10 g L^−1^ glucose to allow cofactor regeneration and incubated with 1 mmol of **7**. After 48 h, 29 % of the substrate was converted into **8**, yielding 59 mg of the pure product, corresponding to an overall yield of 19.4 %.

**Scheme 5:**
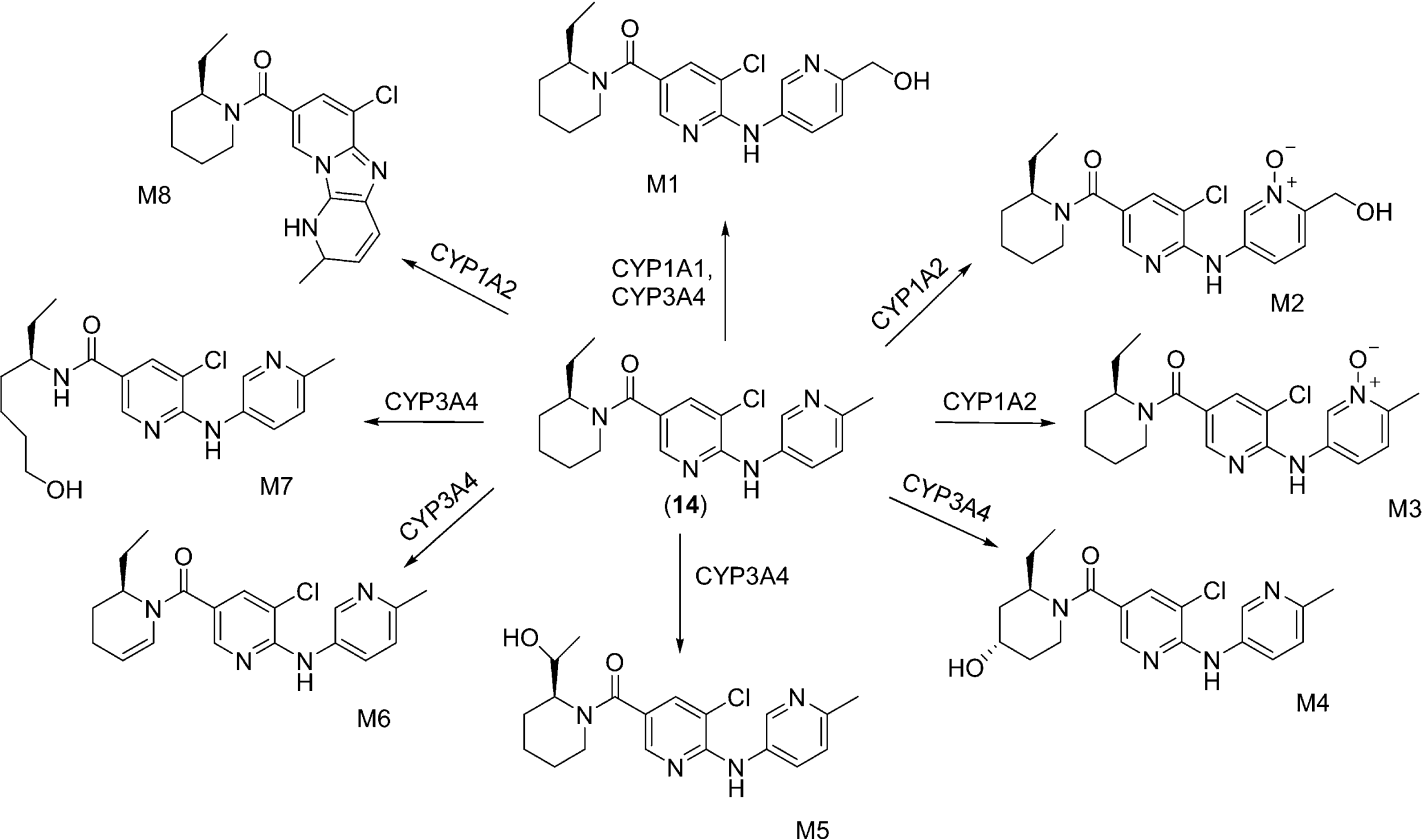
Metabolites of a mGlu5 receptor antagonist (**14**) generated by human CYP isoforms.

An *E. coli* based platform consisting of the 14 major human CYP isoforms has been generated at the University of Dundee, Scotland, in the course of a collaboration project with pharmaceutical companies (LINK Program). These strains are nowadays routinely used in the pharmaceutical industry for screening purposes, but also for preparative syntheses on a scale of up to several hundred milligrams of drug metabolites.^32^ For example, these strains were employed to obtain oxidized metabolites of an mGlu5 receptor antagonist (**14**).^33^ The human isoforms CYP1A1, CYP1A2, and CYP3A4 were identified as being responsible for the metabolism of this piperidyl amide based compound to give several different metabolites (Scheme 5). Whole‐cell conversions of 262 mg of **14** were conducted with the CYP isoforms on 2.5 L scale in a wave bioreactor. CYP3A4‐ and CYP1A2‐catalyzed conversions resulted mainly in the formation of M1 and M5 (29.6 mg and 46.4 mg, respectively), while the other metabolites were produced in amounts less than 10 mg.

**Scheme 6:**
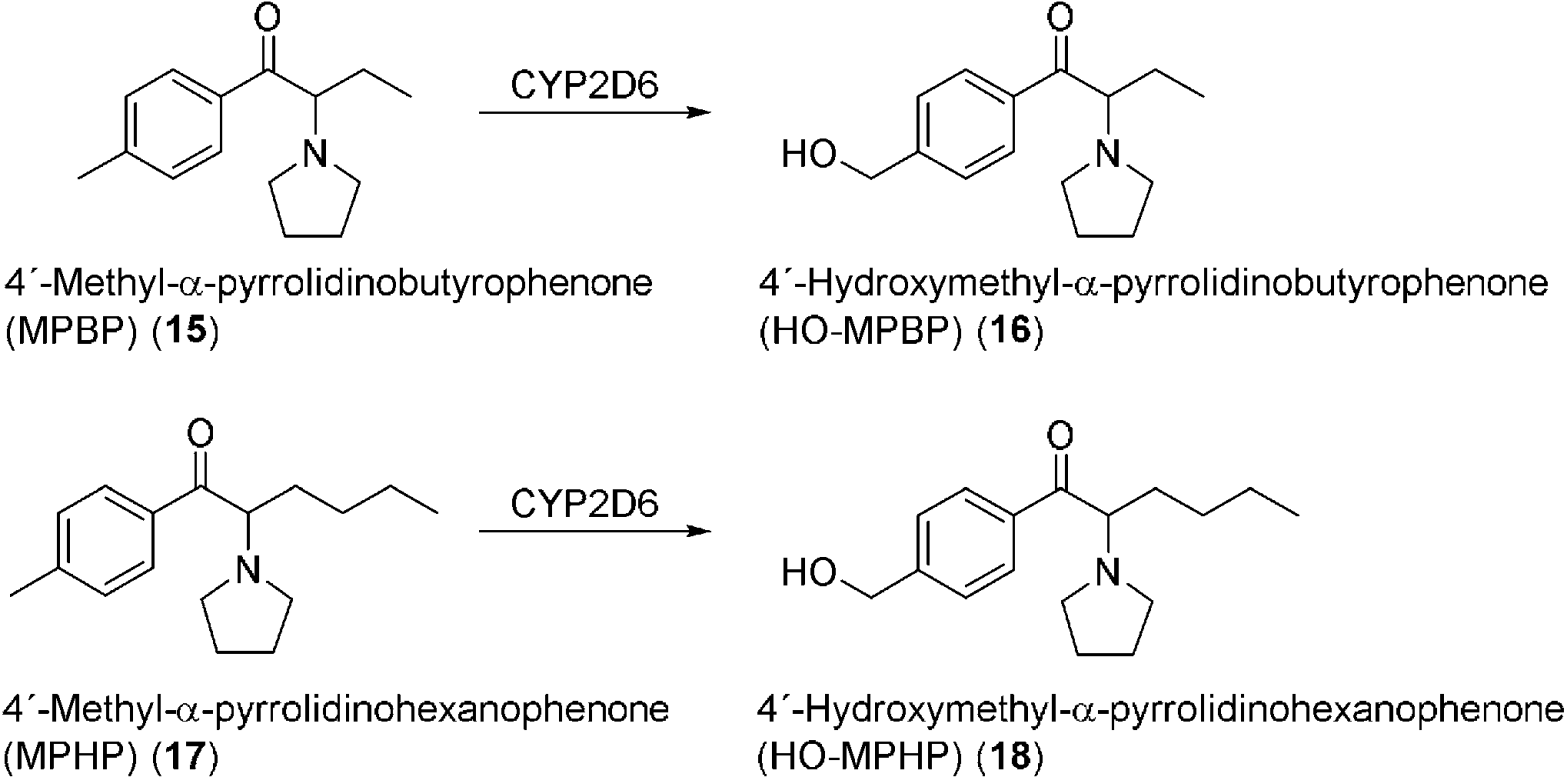
Biooxidation of the 4′‐methyl substituted pyrrolidinophenones **15** and **17** by human CYP2D6.

Several examples show that the fission yeast *Schizosaccharomyces pombe* is not only an efficient expression host for human CYPs, but that corresponding recombinant strains can be easily exploited for the biotechnological synthesis of drug metabolites. Metabolites of the pyrrolidinophenone‐type designer drugs 4′‐methyl‐α‐pyrrolidinobutyrophenone (MPBP, **15**) and 4′‐methyl‐α‐pyrrolidinohexanophenone (MPHP, **17**) were synthesized by fission yeast expressing human CYP2D6^34^, ^35^ (Scheme 6). For biotransformations, 250 μm of the corresponding substrate were incubated with 1 L of fission yeast culture (10^8^ cells mL^−1^). In case of **15**, full conversion to the 4′‐hydroxylated metabolite (HO‐MPBP, **16**) was achieved within 48 h. 40 mg (141 μmol, 56 % yield) of **16** in more than 98 % purity were finally isolated.^34^ Additionally, **17** was fully converted after 66 h. Besides the desired metabolite **18**, two non‐identified byproducts were also observed. **18** was isolated as its hydrochloride salt with an overall yield of 55 % (43 mg, 138 μmol) and more than 99 % purity.^35^ Notably, the described biotechnological approach required a single step for metabolite production, while chemical hydroxylation of the MPBP and MPHP homologue pyrovalerone involved eight different reaction steps, some of which require harsh reaction conditions and hazardous reagents.^36^

**Scheme 7:**
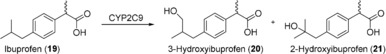
Ibuprofen **19** is metabolized by human CYP2C9 to 3‐hydroxyibuprofen (**20**) and 2‐hydroxyibuprofen (**21**).

Ibuprofen (**19**) is an NSAI drug that is mainly converted into 3‐hydroxyibuprofen, 3‐carboxyibuprofen, and 2‐hydroxyibuprofen during Phase I metabolism. While the latter two compounds are readily accessible through chemical synthesis, an efficient synthesis route for the 3‐hydroxylated metabolite had not been available. CYP2C9 was shown to be involved in the clearance of ibuprofen in the human body, catalyzing its 2‐ and 3‐hydroxylation (Scheme 7) as well as the subsequent 3‐oxidation.^37^, ^38^ Consequently, an *S. pombe* whole‐cell catalyst expressing CYP2C9 and human CPR was used for the biotransformation of **19** (1 mm) on a 1 L scale for 75 h.^39^ The production rates achieved for 3‐hydroxyibuprofen (**20**) exceeded those for 2‐hydroxyibuprofen (**21**; 125 μmol L^−1^ d^−1^ and 44 μmol L^−1^ d^−1^), resulting in 44 mg of isolated and purified **20** (20 % of the theoretical yield).

Besides the reported examples, a platform of human CYPs expressed in fission yeast was established by the biotech company PomBioTech, which exploited it commercially for the production of custom‐made metabolites.

**Scheme 8:**
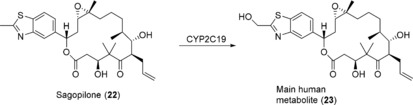
Biooxidation of sagopilone (**22**) to its main metabolite by CYP2C19.

In a recent example, the production of the main human metabolite of sagopilone (**22**) was achieved on a multigram scale.^40^ Sagopilone is an epothilone analogue and is currently being tested in clinical trials as an antitumor agent. Recombinant *E. coli* co‐expressing CYP2C19 and CPR was employed as catalyst for the biooxidation in a resting‐cell biotransformation. Optimization of the fermentation step that produces the biocatalyst as well as of the actual biotransformation step enabled efficient metabolite production on large scale (100 L). The biotransformation of 9 g of **22** resulted in complete conversion to its hydroxylated derivative **23** within 23 h (Scheme 8), which was obtained in analytically pure form in 54 % isolated yield (5.03 g).

**Scheme 9:**
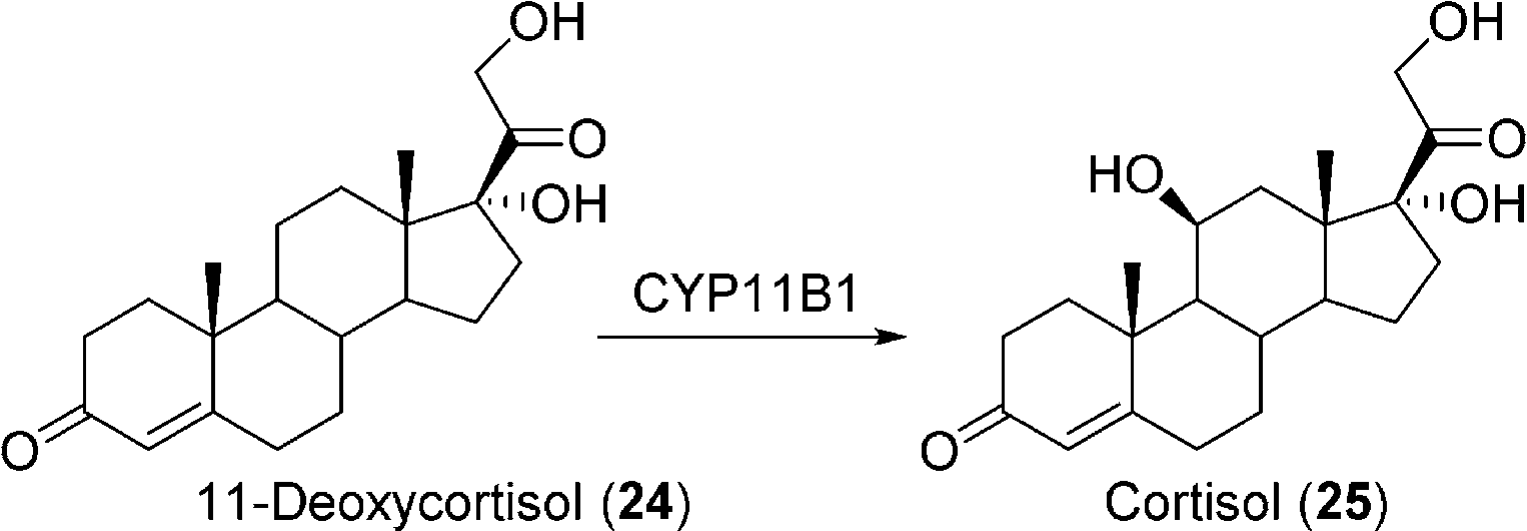
Selective 11β‐hydroxylation of **24** to cortisol (**25**) catalyzed by human CYP11B1.

Besides their key role in human drug metabolism, CYPs are also involved in the steroid biosynthesis pathway of the adrenal gland and are therefore interesting biocatalysts for the selective decoration of steroid backbones. The mitochondrial CYP11B1 catalyzes the last step in cortisol biosynthesis, that is, the regio‐ and stereoselective 11β‐hydroxylation of 11‐deoxycortisol (**24**; Scheme 9).^41^ Cortisol (**25**) is not only the major human glucocorticoid and stress hormone, but is also of high pharmaceutical importance because of its anti‐inflammatory and immunosuppressive properties. Current industrial processes for the production of **25** rely on microbial transformations. For example, **24** is converted with fungi belonging to the genus *Curvularia* by exploiting the endogenous activity of a fungal CYP.^42^, ^43^ However, undesirable side reactions occur due to the low regioselectivity of the corresponding enzyme, which decrease the economic efficiency of the microbial production process. Thus, attempts were made to set up efficient biocatalysts based on the human CYP11B1. In first examples, fission yeast was employed to recombinantly produce CYP11B1 and to convert **24** into **25**.^44^, ^45^ When the redox partners adrenodoxin and adrenodoxin reductase were co‐expressed, a production efficiency of **25** of 1 mm in 72 h was achieved.^45^ A CYP11B1‐based whole‐cell system was also established for the synthesis of **25** in *E. coli*.^46^ In bioconversions of **24** with non‐growing cells in buffer supplemented with glycerol as a carbon source for NADPH regeneration, a volumetric productivity of 843 mg L^−1^ d^−1^ was achieved. **25** can also be produced from simple carbon sources such as ethanol and glucose by employing engineered *S. cerevisiae*.^47^ The yeast metabolism was engineered such that it is capable of mimicking mammalian **25** biosynthesis. For this purpose, four CYPs were introduced into yeast including the human CYP11B1 and CYP21A1. A successful process for the production of cortisol (**25**, hydrocortisone) was developed based on such an engineered yeast strain.^48^

**Scheme 10:**
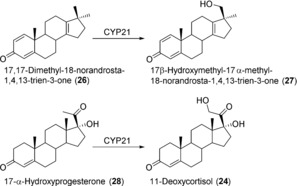
Steroid hydroxylations catalyzed by human CYP21.

The steroidogenic CYP21 was used to produce a metabolite of the anabolic steroid metandienone with a long half‐life, which is required as a reference compound to prove abuse of this doping agent.^49^ A whole‐cell catalyst based on CYP21‐producing *S. pombe* was used to transform 17,17‐dimethyl‐18‐norandrosta‐1,4,13‐trien‐3‐one (**26**), which is chemically derived from metandienone, into the desired metabolite 17β‐hydroxymethyl‐17α‐methyl‐18‐norandrosta‐1,4,13‐trien‐3‐one (**27**), also referred to as 20OH‐NorMD (Scheme 10). The biotransformation of 200 μm of **26** was conducted on a 5 L scale for approximately 92 h. Product extraction and purification resulted in 10 mg of **27**. In addition, 10 mg of a byproduct were isolated, which was identified to be the C16 β‐hydroxylated metabolite. CYP21 produced in *S. pombe* has also been employed for the selective hydroxylation of 17α‐hydroxyprogesterone (**28**) to **24**. Using permeabilized resting cells under optimized conditions resulted in a productivity of 540 μmol L^−1^ d^−1^.^50^

**Scheme 11:**
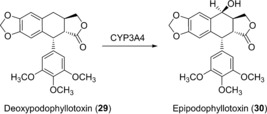
Hydroxylation at the C7‐position of **29** to epipodophyllotoxin (**30**) catalyzed by human CYP3A4.

Recently, it has been shown that plant‐specific biosynthetic steps can be mimicked by human CYPs. CYP3A4 is able to perform the hydroxylation of deoxypodophyllotoxin (**29**) at position C7 (Scheme 11).^51^ These bioconversions have potential for the industrial production of epipodophyllotoxin, which is the aglycon and thus an important building block of the very potent clinical antitumor agents etoposide and teniposide, which are used in the treatment of small‐cell lung cancer and Kaposi's sarcoma.^52^, ^53^, ^54^

Human CYPs can also replace plant enzymes in the biosynthesis of the benzylisoquinoline alkaloid morphine. Although not strictly a hydroxylation, human CYP2D6 was shown to catalyze the oxidative phenol coupling reaction to yield salutaridine from reticuline,^55^, ^56^ as well as the demethylation of codeine to morphine.^57^

**Scheme 12:**
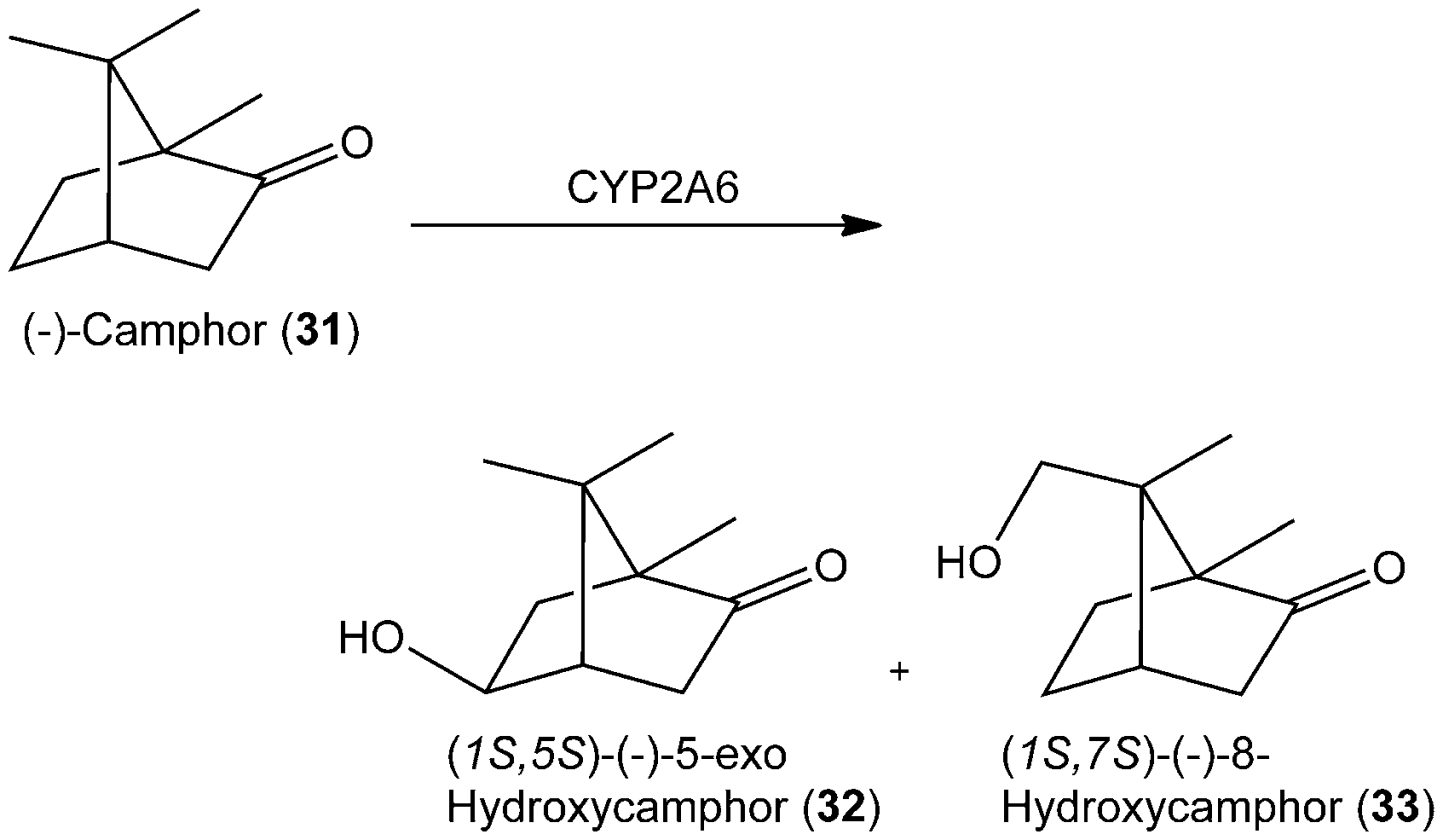
Biotransformation of (−)‐camphor (**31**) by human CYP2A6.

Finally, the hydroxylation of terpenoids with human CYPs has also been conducted on a preparative scale.^58^ CYP2A6 and CPR were co‐expressed in *Salmonella typhimurium* and used in whole‐cell conversions of (−)‐camphor (**31**). Biotransformation of 400 mg substrate resulted in the isolation of 30.4 mg (1*S*,5*S*)‐(−)‐5‐*exo*‐hydroxycamphor (**32**) and also 2.4 mg (1*S*,7*S*)‐(−)‐8‐hydroxycamphor (**33**; Scheme 12), whereas the bacterial P450cam showed selectivity towards formation of **32**.^59^

### 2.3 Dealkylation

**Scheme 13:**
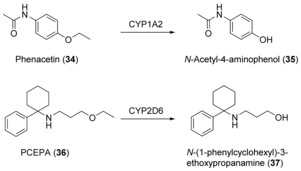
CYP‐mediated de‐ethylations of active pharmaceutical ingredients.

A special case of hydroxylation reactions is hydroxylation in the α position to a heteroatom. In case of a methyl ester, for example, hydroxylation of the methyl group results in an unstable hemiacetal that decomposes to finally yield a demethylation product. O‐Demethylation reactions catalyzed by human CYPs have been documented,^57^, ^60^ however, they have not been utilized on a preparative scale yet. For de‐ethylation of phenacetin (**34**; Scheme 13), *E. coli* expressing a truncated soluble version of human CYP1A2 and its reductase was used as described above for **1** and **7** hydroxylation. After 48 h, 88 mg of pure **35** was isolated.^21^ Similarly, the fission yeast *Schizosaccharomyces pombe* expressing CYP2D6 was used to prepare *N*‐(1‐phenylcyclohexyl)‐3‐hydroxypropanamine (**37**) from the designer drug *N*‐(1‐phenylcyclohexyl)‐3‐ethoxypropanamine (**36**). Although the reaction time was prolonged (72 h), the product yield was only 6 mg, corresponding to 9 % overall yield.^61^

**Scheme 14:**
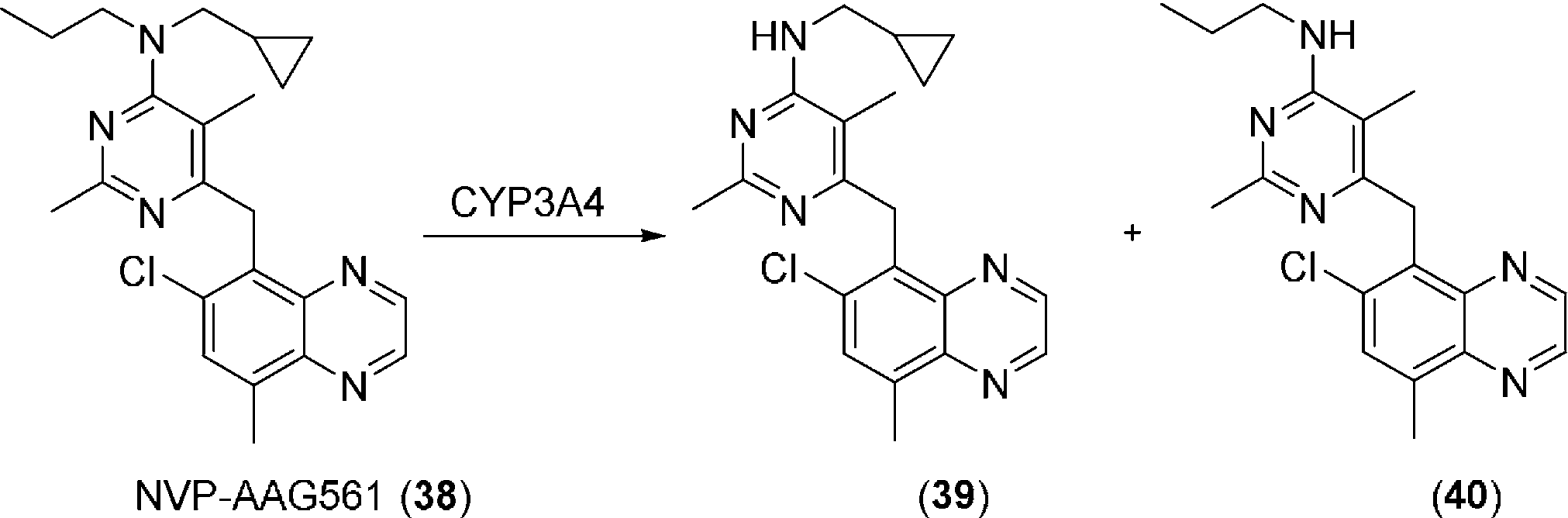
N‐Dealkylations catalyzed by CYP3A4.

In the case of amine‐containing compounds such as the drug candidate NVP‐AAG561 (**38**), dealkylation products **39** and **40** (Scheme 14) were the major compounds isolated from a compound mixture that also included hydroxylated product.^62^ In the case of CYP3A4‐catalyzed oxidation of diazepam, the major metabolite was a hydroxylated compound with the N‐demethylation product being produced on a sub‐milligram scale, as described for **1** and **10**.^20^

**Scheme 15:**
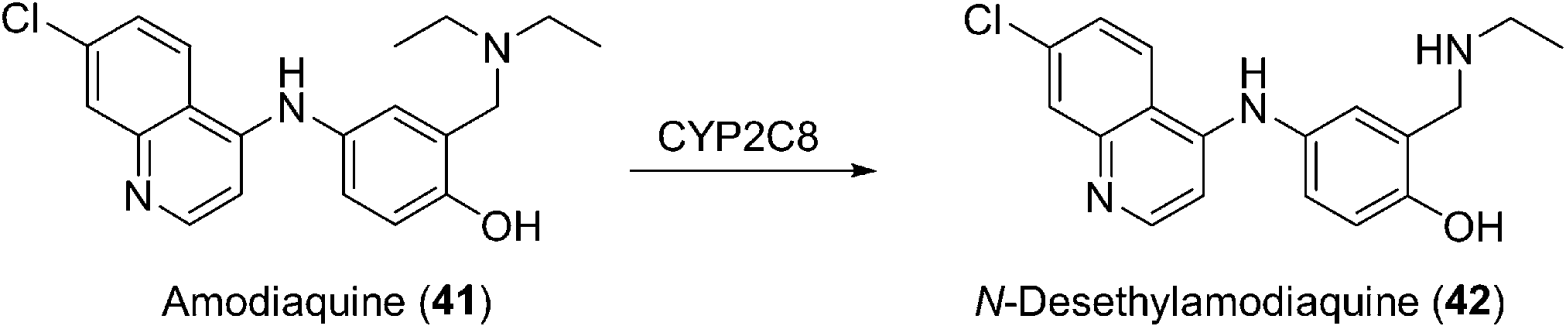
N‐Deethylation of amodiaquine (**41**) catalyzed by CYP2C8.

In the metabolism of amodiaquine (**41**), an antimalarial drug, a hemiaminal is formed that decomposes to the deethylated compound *N*‐Desethylamodiaquine (**42**; Scheme 15). A preparative biotransformation was carried out on a 2 L scale using whole cells of *E. coli* expressing a CYP2C8 variant. **41** was treated for 32 h and 172 mg of **42** was isolated, representing an overall yield of 55 %.^62^

Despite their great synthetic potential, the use of native human CYPs is still somewhat limited. This is mainly due to the complex nature of these enzymes as well as due to their low activity and stability. Protein engineering has been successfully used to address these issues, improving the properties of CYPs for practical applications.^63^, ^64^, ^65^, ^66^ From a preparative perspective, the promiscuity of human CYPs is of course a limitation that demands elaborate product purification steps, which are undesired in view of synthetic applications in general. In the context of drug metabolite synthesis, however, this diversity may well be an advantage.

## 3 Heteroatom oxidation

### 3.1 N‐Oxidation

Molecules containing the soft nucleophile nitrogen are mostly oxidized by flavin monooxygenase enzymes to the corresponding N‐oxides, nitrones, or oximes,^14^ although human CYPs have also been reported to catalyze N‐oxidations.^18^ Flavin monooxygenases are membrane‐associated flavin‐containing proteins that catalyze the oxidation at the expense of NADPH and molecular oxygen. Six human FMO isoforms have been described to date and they are expressed in different human tissues. Isoform FMO3 is the most abundant non‐CYP drug‐metabolizing enzyme variant. Like human CYPs, FMOs exhibit overlapping substrate specificities, however, in comparison to CYPs, FMO enzymes are less complex at the molecular level. They are self‐sufficient in the catalytic cycle and only require the presence of sufficient amounts of NADPH and oxygen but no additional proteins.

**Scheme 16:**
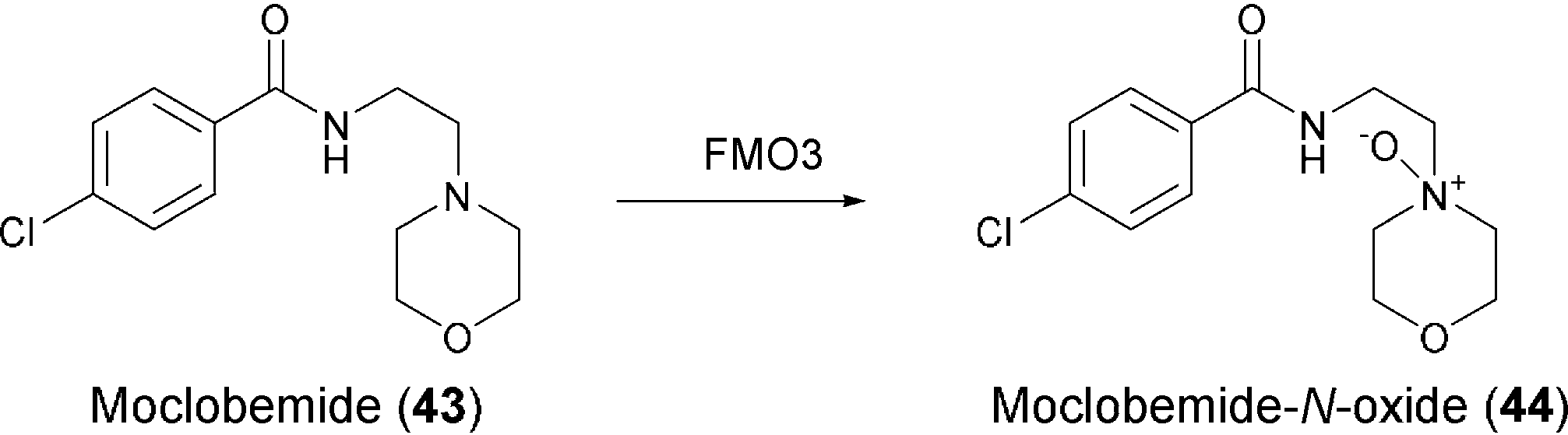
N‐Oxidation of **43** catalyzed by FMO3.

The synthetic utility of human FMOs was demonstrated by the preparation of Moclobemide‐*N*‐oxide (**44**): 100 mg of moclobemide (**43**), an antidepressant acting by monoamine oxidase A inhibition, was converted into **44** in a whole‐cell biotransformation with hFMO3 expressed in *E. coli* BL21. The biocatalyst had been frozen and thawed and the reaction proceeded in shake flasks with 20 % w/v of biocatalyst in the presence of citrate, catalytic amounts of NADP^+^, and air. After 24 h, the product was isolated and purified to give 65 mg of **44** (Scheme 16).^67^

**Scheme 17:**
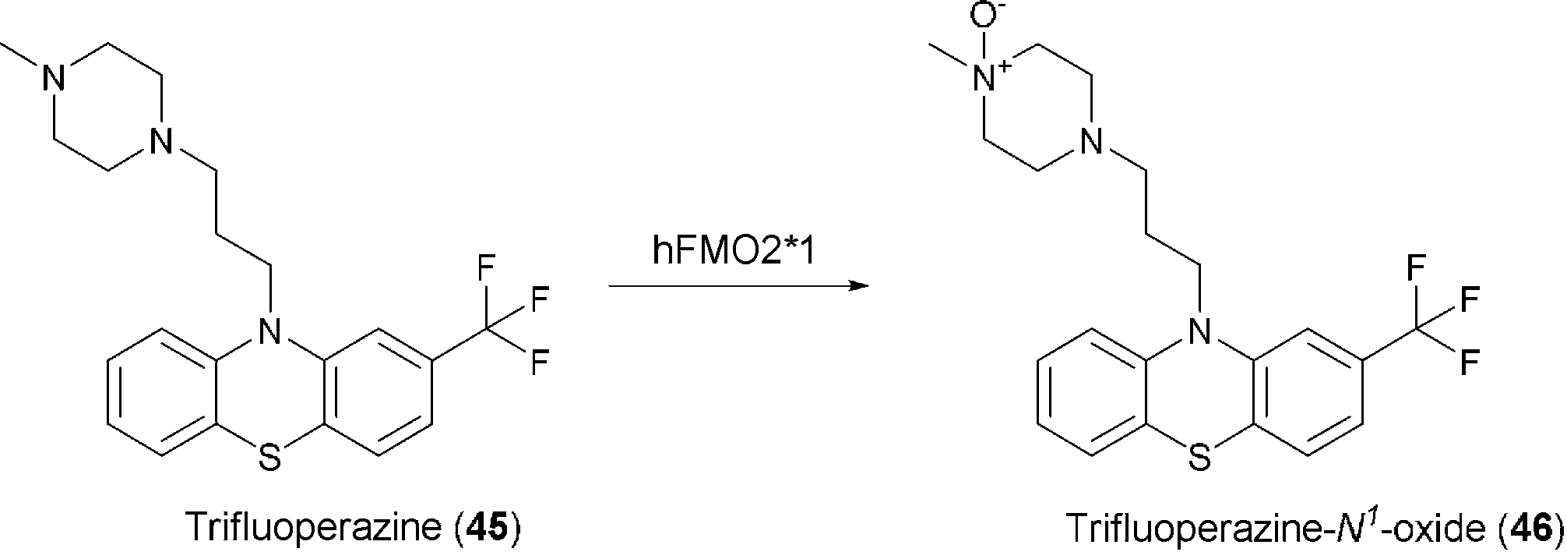
Chemo‐ and regioselective N^*1*^‐Oxidation of **45** catalyzed by FMO2*1.

Isoenzyme FMO2*1 was significantly less well expressed by *E. coli* than FMO3 under identical conditions. The selective oxidation of trifluoperazine (**45**), an antipsychotic drug, however, proved the cells to be a valuable biocatalyst. Although five soft nucleophiles are present as potential oxidation sites, only the ^*1*^N position of the piperazine ring was oxidized (Scheme 17). Metabolite **46**, an authentic human metabolite, is not accessible by chemical oxidation with either hydrogen peroxide or Na^+^‐periodate, which afforded mixtures of different products.^68^ Moreover, unpublished results from our labs with a truncated FMO4 variant^69^ showed that this isoform was less selective and gave a mixture of different trifluoperazine metabolites.

### 3.2 Other Heteroatom Oxidations

The oxidation of sulfides can be catalyzed by human CYPs or flavin monooxygenase enzymes.^70^ Sulfoxidation leads, in the first step, to a chiral sulfoxide and its further oxidation to the sulfinic acid. Human FMOs can also catalyze the oxidation of selenium, in contrast to human CYPs.^71^ Preparative applications of human CYPs or FMOs may become valuable in cases where enantio‐, chemo‐ or regioselectivity are required.

## 4 Other Oxidations

### 4.1 C−O Bond Oxidation

**Scheme 18:**
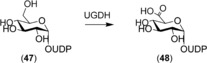
UGDH‐catalyzed oxidation of primary sugar alcohol.

The repertoire of alcohol dehydrogenases in humans is mainly related to the oxidative metabolism of ethanol and retinol to the respective aldehydes.^72^ ADH enzymes from various other sources have been extensively studied, and are abundant and cheaply available. In contrast to some selective keto‐reductases, human ADH enzymes have not played an important role in synthetic applications yet. One exception is the selective oxidation of uridine‐diphosphate glucose (UDP‐glucose, **47**) with human UDP‐glucose‐6‐dehydrogenase (UGDH, **48**) to the corresponding UDP‐glucuronic acid (Scheme 18). The enzyme was expressed in *Schizosaccharomyces pombe* and used in the form of permeabilized whole cells, and a maximum of 7.2 mm of the UDP glucuronic acid was formed in the presence of 2 equivalents of NAD^+^ and 24 g L^−1^ of dry cells within 5 h. Product isolation was not reported.^73^

### 4.2 C−N Bond Oxidation

In the human body, primary amines such as the neurotransmitters serotonin, dopamine, and epinephrine are oxidized by monoamine oxidase enzymes (MAOs) to the respective imines, which subsequently decompose to the respective aldehydes in the aqueous environment.^74^ In addition, the oxidation of tertiary amines has been described, for example, to generate cyclic iminium ions.^75^ Although the latter reaction is useful for the preparation of chiral amines,^76^ preparative‐scale reactions employing these human mitochondrial enzymes have not been reported yet.

### 4.3 Baeyer–Villiger Monooxygenations

**Scheme 19:**
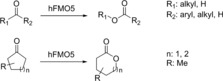
FMO5‐catalyzed oxygenation of aliphatic and cyclic carbonyl compounds.

Baeyer–Villiger monooxygenases (BVMOs) catalyze oxidation reactions in which carbonylic compounds are converted into the corresponding esters or lactones. These products are important building blocks in organic and polymer syntheses. The synthetic potential of BVMOs has broadly been illustrated for enzymes of microbial origin.^77^ The human genome lacks the presence of a typical BVMO gene.^78^ However, only recently, the human flavin‐containing monooxygenase isoform 5 (hFMO5) was shown to act as a BVMO on a broad range of substrates.^79^, ^80^ While hFMO5 is non‐ or poorly active on standard FMO substrates with soft nucleophiles, aliphatic and cyclic ketones such as 6‐methylhept‐5‐en‐2one or phenylacetone were converted efficiently (Scheme 19). These conversions were performed with purified enzyme and substrate concentrations of 5 mm.^80^ The potential of hFMO5 for preparative syntheses still needs to be investigated.

### 4.4 Epoxidation

**Scheme 20:**
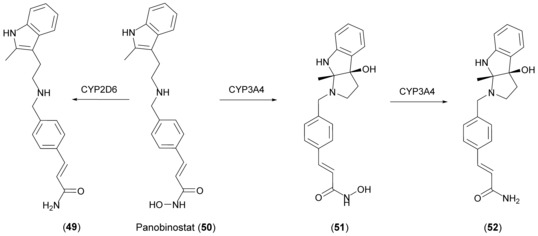
Metabolic reactions on **50**. CYP3A4 mediates epoxidation followed by intramolecular cyclization to **51**. Reductive conversion of the hydroxamic acid to the respective amide is catalyzed by both CYP3A4 and CYP2D6.

Like other cytochrome P450 enzymes, human variants have also been shown to catalyze epoxidation reactions, such as the epoxidation of lipid molecules.^81^ On a synthetic scale, this reaction was utilized to prepare panobinostat (**50**) metabolites: CYP3A4 forms an epoxide intermediate that undergoes an intramolecular cyclization reaction. Unlike the epoxide intermediate, 5 mg of **51** could be isolated from a bioconversion of 205 mg of panobinostat lactate (Scheme 20).^82^

## 5 Reductions

### 5.1 Carbonyl Reduction

**Scheme 21:**
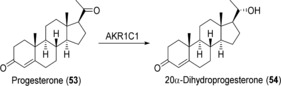
AKR1C1‐catalyzed reduction of progesterone (**53**).

The preparation of steroid derivatives for use as drugs is of great interest in the pharmaceutical industry and, therefore, enzymes which modify these compounds are potentially interesting biocatalysts. Human aldo‐keto reductases (AKRs) have recently come to prominence in this regard, an example being the preparation of 20α‐dihydroprogesterone (**54**) through reduction of the 20‐keto group of progesterone (**53**; Scheme 21) using 20α‐hydroxysteroid dehydrogenase (AKR1C1).^83^ Chemical reduction of such hydroxysteroids typically results in the formation of the β‐isomer,^84^ therefore increasing the need for a stereoselective biocatalytic process. AKR1C1 was expressed in the fission yeast *S. pombe* and the whole‐cell biocatalyst produced **54** in good yield. Although the substrate scope was found to be rather limited, with only progesterone and the synthetic progesterone analogue dydrogesterone (**55**) showing product formation out of 9 steroids tested, the activity was higher with **55** than with the native substrate. In a 1 L whole‐cell fed‐batch process, 310 mg of **53** (1 mm) was converted into **54** in 72 h (biotransformation yield 90 %) and 30 mg of pure product could be isolated. The relatively low overall yield was attributed to a non‐optimized product isolation procedure.

**Scheme 22:**
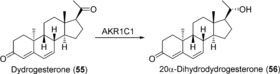
AKR1C1‐catalyzed reduction of dydrogesterone (**55**).

In a subsequent publication,^85^ impressive improvements were made to the system, thereby allowing the preparation of 20α‐dihydrodydrogesterone (**56**; Scheme 22) in multigram amounts on a pilot scale. The key changes were modification of the expression plasmid to include four expression units instead of one, the use of cyclodextrin to improve substrate solubility, and the optimization of product purification. Volumetric productivity was increased 42‐fold compared to the original procedure, and from 30 L reactions lasting for 136 h to which 20 g of **55** was added, an average of 12.3 g of **56** was isolated in 63 % overall yield. In addition, the same process was used to prepare 2.5 g of **54**.

**Scheme 23:**
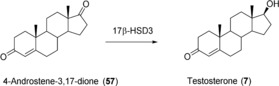
17β‐Hydroxysteroid dehydrogenase mediated reduction of **57** to testosterone (**7**).

Using codon‐optimized human 17β‐hydroxysteroid dehydrogenase type 3 (17β‐HSD3) co‐expressed with *S. cerevisiae* glucose‐6‐phosphate dehydrogenase in *Pichia pastoris*, **7** was obtained from 4‐androstene‐3,17‐dione (**57**). In this case, whole cells (200 g L^−1^ wet cell weight) were used for preparative reactions, with 5 g L^−1^ of **57**. The reaction was carried out for 48 h. A total of 4.56 g L^−1^ of **7** was detected, corresponding to a productivity of 95 mg L^−1^ h^−1^ (Scheme 23).^86^

**Scheme 24:**
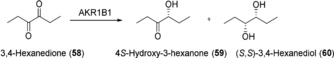
AKR1B1‐catalyzed reduction of 3,4‐hexanedione (**58**).

**Scheme 25:**
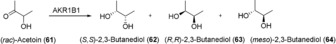
AKR1B1‐catalyzed reduction of acetoin (**61**).

The preparation of α‐hydroxyketones and vicinal diols for use as chiral synthetic intermediates via reduction of diketones is of great interest to organic chemists.^87^ Two human aldo‐keto reductases (AKR1B1 and AKR1B10) were investigated in this regard and compared with three similar enzymes from yeast.^88^ The enzymes were expressed in *E. coli* and subsequent analytical scale experiments carried out using purified proteins. Preparative examples were not presented, however, the human enzymes exhibited some distinct characteristics; for example, in the reduction of 3,4‐hexanedione (**58**; Scheme 24), AKR1B1 was the only enzyme capable of reducing both keto groups yielding (3*S*,4*S*)‐3,4‐hexanediol (**60**), although the main product was *S*‐4‐hydroxy‐3‐hexanone (**59**). In the case of acetoin (**61**), both human and yeast enzymes produced a mixture of diols, but only AKR1B10 was capable of producing (*2R*,*3R*)‐2,3‐butanediol (**63**), albeit in small amounts (Scheme 25).

### 5.2 Other Reductions

The promiscuity of human CYPs includes the reductive conversion of hydroxamic acids to the respective amides. Fredenhagen et al. utilized CYP2D6 and CYP3A4 to produce metabolites of **50**. Using CYP2D6, 268 mg of panobinostat amide (**49**) were isolated and with CYP3A4, 12 mg of **52** were purified from a mixture with **51** (Scheme 20).^82^

### 5.3 Disproportionation Reactions

**Scheme 26:**
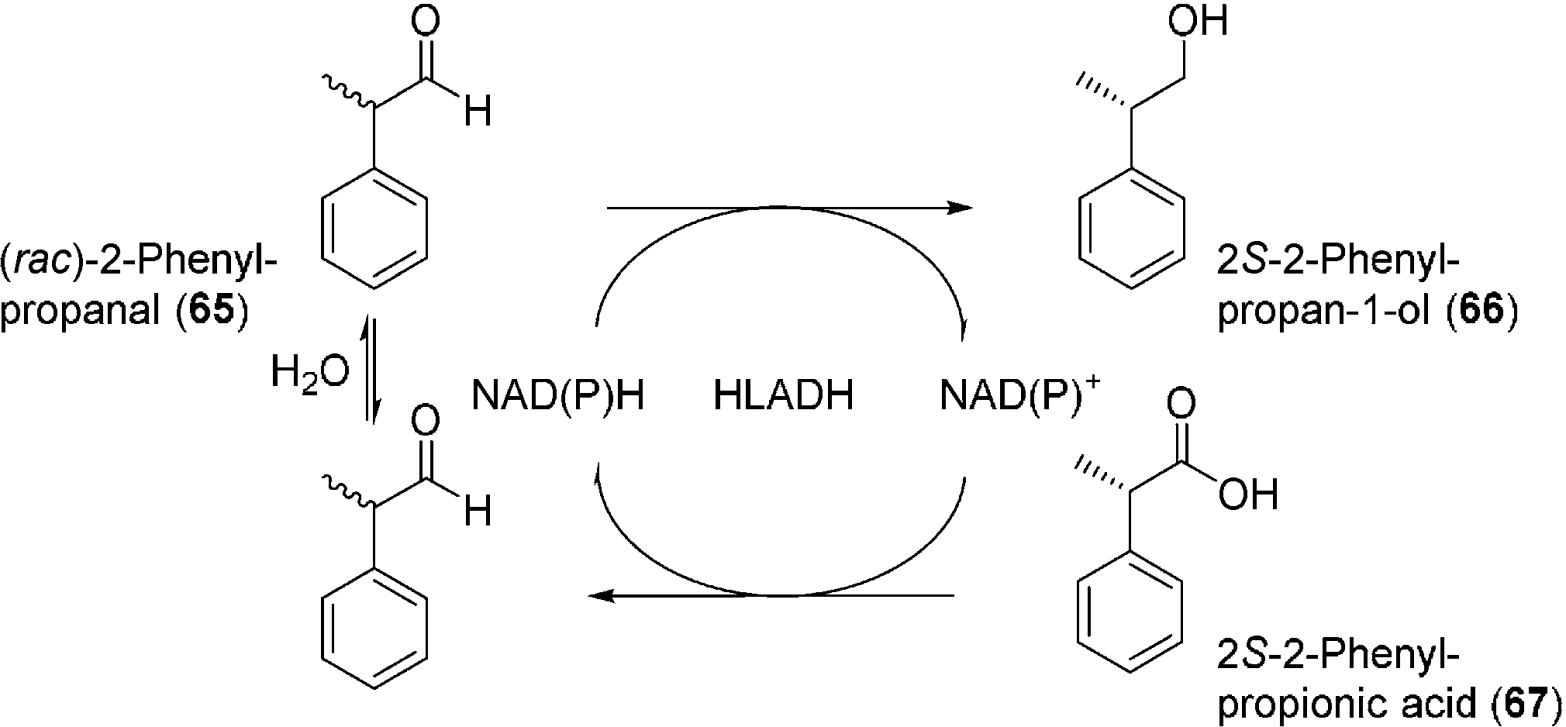
HLADH‐catalyzed disproportionation of *rac*‐2‐phenylpropanal (**65**).

Biocatalytic Cannizzaro‐type reactions, namely disproportionation of aldehydes to form the corresponding alcohols and carboxylic acids, have been described using human liver alcohol dehydrogenase (HLADH) in a comparison with several microbial enzymes.^89^ Initially, two substrates, benzaldehyde and phenylacetaldehyde, were tested. At 10 mm concentration, the performance of HLADH was rather moderate, with conversions of 13 and 26 %, respectively. However, when the feasibility of an asymmetric Cannizzaro reaction was investigated using 10 mm *rac*‐2‐phenylpropanal (**65**), HALDH displayed by far the best selectivity of the enzymes tested, producing the *S*‐2‐phenylpropan‐1‐ol (**66**) in 99 % enantiomeric excess (*ee*) and the *S*‐2‐phenylpropionic acid in 87 % *ee* (**67**) (Scheme 26), although conversion was again low (12 %). Increasing the enzyme loading from 0.5 to 5 mg mL^−1^ allowed almost complete conversion (97 %) and led to a more balanced ratio of products. The redox‐neutral nature of the process, with each half‐reaction generating the cofactor necessary for the other (Scheme 26), and the spontaneous racemization of the aldehyde substrate leading to a dynamic resolution are attractive aspects of this reaction. It remains to be seen whether further improvements can be made that will facilitate application on a preparative scale.

## 6 Hydrolytic Reactions

The enzymatic addition of water to substrates that are susceptible to hydrolysis is catalyzed by a number of different hydrolytic enzymes in the human body. Hydrolases play an important role not only in the metabolism of nutrients, but also in drug metabolism.^90^ To mention one selected example, the methyl ester of one of the most extensively prescribed drugs, clopidogrel and its CYP‐oxidized metabolites, is cleaved by carboxylesterase 1 to the respective carboxylic acid. Other hydrolytic reactions include the hydrolysis of lactones, inorganic esters, amides, lactams, and epoxides.^10^ Although a significant number of reports on various aspects of human hydrolases are available, their utility for synthetic applications seems to be limited or has not yet been investigated, with the exception of epoxide hydrolysis.

**Scheme 27:**
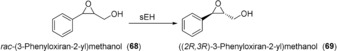
Human sEH‐catalyzed kinetic resolution of racemic epoxide **68**.

Epoxide hydrolases catalyze the hydrolysis of epoxides to form vicinal diols. The soluble mammalian enzymes are members of the α,β‐fold hydrolase family^91^ and are responsible for the hydrolysis of xenobiotic^92^ as well as endogenous epoxides.^93^ Recombinant human soluble epoxide hydrolase (sEH) was compared with enzymes from mouse and cress for utility in the regio‐ and enantioselective hydrolysis of phenyloxiranes such as *rac*‐(3‐phenyloxiran‐2‐yl)methanol (3‐phenylglycidol, **68**; Scheme 27).^94^ The enzyme was expressed in a *Trichoplusia ni* baculovirus system and was purified prior to use. Although the selectivity was modest with all the compounds tested (E values 1–7), a preparative reaction was carried out which contained 150 mg of **68** and 15 mg purified sEH in 100 mL phosphate buffer (pH 7.4). The reaction was stopped after 8 h (approx. 70 % conversion) and 46 mg of the retained epoxide with an *ee* of 94 % was obtained after purification.

## 7 Glycosylation Reactions

The turnover of glycosides in human cells reflects the interplay of synthetic glycosyltransferase (Enzyme Commission number EC 2.4.) and degradative glycoside hydrolase (EC 3.2.) activities under the constraints of subcellular compartmentation. Glycosyltransferases utilize an activated glycosyl donor, typically a nucleotide sugar, to glycosylate an acceptor molecule, usually with high chemo‐, diastereo‐, and regioselectivity. Nine nucleotide sugars are the natural substrates of human glycosyltransferases: uridine 5′‐diphospho (UDP)‐α‐d‐glucose, UDP‐α‐d‐galactose, UDP‐α‐*N*‐acetyl‐d‐glucosamine, UDP‐α‐*N*‐acetyl‐d‐galactosamine, UDP‐α‐d‐xylose, UDP‐α‐d‐glucuronic acid (UDP‐GlcA), guanosine 5′‐diphospho (GDP)‐α‐d‐mannose, GDP‐β‐l‐fucose, and cytidine 5′‐monophospho‐β‐*N*‐acetyl‐neuraminic acid.^95^ Glycoside synthesis has been performed mainly with UDP‐GlcA dependent glucuronosyltransferases.^96^

**Scheme 28:**
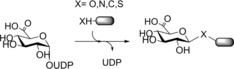
General scheme of glucuronidation reactions catalyzed by human UGTs.

Note that the focus here is on small‐molecule glycosides. Not considered are glycoproteins, glycolipids, and oligo‐ and polysaccharides such as heparins and other glycosaminoglycans. The application of human enzymes for in vitro synthesis and processing of glycan structures has so far been limited to the glycoengineering of proteins.^97^ Nevertheless, the human metabolism of small molecules through glycosylation by UDP‐sugar‐dependent glycosyltransferases has also attracted particular attention.^96^, ^98^, ^99^, ^100^ Reported syntheses have typically been performed on a small scale (≤mg). The principal role of glucuronidation in human phase II drug metabolism explains the strong focus on glucuronosyltransferase enzymes and their reactions with various acceptor substrates (Scheme 28).^96^, ^99^ Besides working strictly one after the other in drug metabolism, CYPs and UGTs can also compete for substrates. Compounds glucuronidated early, or prematurely, in metabolism may even act as inhibitors of CYPs.^101^ This emphasizes the need to determine UGT reactivity with drug metabolites at all stages of the metabolism.

### 7.1 Glycosyltransferases

Human UDP‐sugar‐dependent glycosyltransferases (hUGT) form a superfamily of enzymes comprising four families, generally referred to as UGT1, UGT2, UGT3, and UGT8.^98^ In the CAZy (Carbohydrate‐Active Enzymes) classification of glycosyltransferases, hUGTs are found in Glycosyltransferase family 1.^102^ Enzymes adopt the so‐called GT‐B fold, which is broadly characterized as consisting of two similar Rossmann‐fold domains that include the active site in the interdomain cleft.^103^ UGT1 and UGT2 are mainly glucuronosyltransferases. UGT3 enzymes differ in donor substrate specificity and utilize UDP‐GlcNAc, UDP‐Glc, or UDP‐Xyl preferentially. UGT8 has been shown to glycosylate ceramide from UDP‐Gal and thus appears to play a role in sphingolipid synthesis. Individual UGTs within a particular family differ in acceptor substrate specificity.^98^ The aglycone substrate specificity of hUGTs in relation to human drug metabolism has been reviewed.^96^, ^99^ hUGTs are membrane‐bound or membrane‐associated proteins and therefore difficult to produce recombinantly and characterize. Use of the enzymes in whole cells (e.g. *Saccharomyces cerevisiae* 104 or *Schizosaccharomyces pombe* 14, 105, 106, 107, 108) appears to be promising for facilitated synthesis, but this remains to be demonstrated in a broader sense.

### 7.2 O‐Glycosylations

Beside the highly prevalent glucuronidation, O‐glycosylation also includes the attachment of a d‐glucosyl, *N*‐acetyl‐d‐glucosamine, or d‐xylosyl residue.^96^, ^98^ For example, morphine is mainly glucuronidated at position 3 or 6 depending on the enzyme used, but modifications with a glucosyl residue have also been found.^98^ Mono‐glycosylation is by far the most important transformation but bis‐ and di‐glycosylations are also possible. During bis‐glycosylation, two sugars become attached to different functional groups of the aglycone. Bilirubin is a compound for which multiple glycosylations have been identified.^98^ A mixed bis‐glycoside results when the attached sugars differ. 17α‐Estradiol‐3‐glucuronide‐17‐*N*‐acetylglucosaminide is an example.^98^ Diglycosides have two sugars linked to each other, only one of which is attached to the aglycone, such as in dihydrotestosterone diglucuronide, for example.^104^ The site selectivity of O‐glycosylation differs strongly among individual human enzymes and varies additionally with the structure of the aglycone.^98^, ^109^, ^110^

The case of morphine glucuronidation is interesting.^98^ The 6‐*O*‐glucuronide is pharmacologically more active than the parent aglycone. The alternative 3‐*O*‐glucuronide is inactive by contrast. Species differ in the extent to which the 6‐*O*‐glucuronide accumulates in addition to the major 3‐*O*‐glucuronide. In humans, 6‐*O*‐glucuronide is found to account for 10–20 % of total morphine.^98^ Synthetic applications remain to be developed for these enzymatic reactions.

Ester‐linked glycosides are formed frequently in the metabolism of human drugs.^98^, ^111^, ^112^, ^113^ Ibuprofen, ketoprofen, naproxen, diclofenac, diflunisal, clofibric acid, and valproic acid are well‐studied examples. They are mostly glucuronidated but other glucosylation has also been shown. Compounds like retinoic acid are likewise glycosylated at the carboxylic acid group.^96^ Mycophenolic acid can be glucuronidated at the hydroxy group and at the carboxylic acid group. The “selectivity” of liver homogenates from different mammals (not that of humans, however) in the glucuronidation of mycophenolic acid has been studied. Only the horse liver homogenate produced the acylglucuronide in amounts comparable to mycophenolic acid 7‐*O*‐glucuronide. Using optimization of reaction conditions, a shift in selectivity to favor formation of the acylglucuronide was obtained and this could be exploited for preparative synthesis, affording 240 mg acylglucuronide and 14 mg of the 7‐*O*‐glucuronide from 450 mg of mycophenolic acid. UGTA10 is assumed to be the enzyme responsible for both glucuronidations.^114^ The drug candidates candesartan and zolarsartan were glucuronidated at the carboxyl group and the resulting glucuronides were prepared enzymatically on a mg scale. Therefore, recombinant human UGT1A3 and 1A8, respectively, were expressed in baculovirus‐infected insect cells. On a 50–80 mL scale, 15–25 mg of sartan was treated with approximately 100 mg of protein in the presence of UDP‐glucuronic acid to afford up to 6.8 mg of product within 2–3 days.^115^

A hydroxylamine‐linked *O*‐glucuronide of *N*‐hydroxy‐2‐acetylaminofluorene was formed by various hUGTs, including UGT1A6 and UGT2B7, which are strongly expressed in the liver. hUGTs (e.g. UGT1A4, UGT1A6, and UGT1A9) may be able to synthesize *N*‐*O*‐glucuronides of benzidines.^96^ *N*‐Carbamoyl glucuronidation was reported for the drug candidate lorcaserin.^116^

Various hUGTs are diastereoselective in the reaction with their acceptor substrates. For example, *S*‐oxazepam is glucuronidated by UGT2B15, whereas *R*‐oxazepam is glucuronidated by UGT2B7 and UGT1A9.^117^ *O*‐Desmethyltramadol, a CYP2D6 metabolite of the analgesic drug tramadol, is glucuronidated by UGTs 1A7‐1A10 with strict selectivity for the 1*R*,2*R*‐diastereomer.^118^ UGT2B7 was similarly selective although not strictly so. UGT2B7 reacted with both diastereomers, showing slight preference for 1*S*,2*S*‐*O*‐desmethyltramadol. UGT2B15 exhibited high *cis* selectivity for glucuronidation of 4‐hydroxytamoxifen.^118^ Well‐known drugs such as naproxen and ibuprofen are glucuronidated with widely varying stereoselectivity by UGTs but UGT1A1 is stereoselective for reaction with both substrates.^96^

### 7.3 N‐Glycosylations

N‐linked glycosylation occurs in the metabolism of various human drugs.^96^, ^98^ Glucuronidation of the antiepileptic drug retigabine was observed at two N sites.^119^ hUGT‐catalyzed glucuronidation of different sartans was studied, including losartan, which is a receptor AT1 antagonist currently in clinical use for treatment of hypertension. Reactions occurred at oxygen but also at different nitrogen atoms of the tetrazole ring. The products were synthesized in mg amounts (0.5–6.8 mg) for characterization.^115^, ^120^ The reactions involved 5 mm of UDP‐GlcA and a variable sartan concentration between 0.25 and 2.00 mm. They were performed for up to 50 h using a protein concentration in the range 0.5–3.5 mg mL^−1^. Recombinant enzymes and liver microsomal preparations were used, affording yields in the range of 1.1–34.6 %. Products were isolated by HPLC with impurities in the range of 0.7–37.1 %.^122^ UGT1A3 was found to be highly selective towards the tetrazole‐N^2^.^115^, ^120^ The N^+^‐glucuronidation of 4‐hydroxy‐tamoxifen was catalyzed by UGT1A4, whereas many other hUGTs catalyzed 4‐*O*‐glucuronidation.^121^ N^+^‐glucuronides of other drugs (e.g. midazolam,^122^ sarpogrelate^123^) were also reported.

### 7.4 C‐ and S‐Glycosylations

Compared to O‐ and N‐glycosylation, C‐glycosylation appears to be rare. Phenylbutazone, for example, undergoes both O‐ and C‐glucuronidation during human metabolism. Among the human hUGTs, UGT1A9 was the only enzyme capable of forming the C‐glucuronide.^124^ Its specific activity was, however, low (7.4 pmol min^−1^ mg^−1^ protein) compared to the specific activities of other hUGTs for O‐glucuronidation of phenylbutazone. UGT1A3 was most active in forming the O‐glucuronide (>100 pmol min^−1^ mg^−1^ protein) but lacked activity for C‐glucuronidation. UGT1A9 showed a specific activity of 1.6 pmol min^−1^ mg^−1^ protein for O‐glucuronidation of phenylbutazone. UGT1A9 also forms the C‐glucuronide of sulfinpyrazone.^125^ Interestingly, C‐glycosylation is relatively widespread in plants and microorganisms, but there, it appears to be restricted to the formation of aryl‐C‐glycosidic linkages.^126^

Bureik and colleagues reported S‐glucuronidation of mercapto‐4‐methylcoumarin by 13 out of the 19 hUGTs on an analytical scale, thus suggesting this activity to be widely distributed among these enzymes.^106^ The cardioselective potassium‐ATP channel blocker HMR1098 has been shown to be S‐glucuronidated by different hUGTs.^127^

## 8 Conjugation Reactions

### 8.1 O‐Methylation

**Scheme 29:**
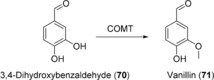
COMT‐catalyzed O‐methylation of 3,4‐dihydroxybenzaldehyde (**70**).

Soluble catechol‐*O*‐methyltransferase (COMT) catalyze the O‐methylation of biogenic amines such as dopamine by using *S*‐adenosyl methionine (SAM) as activated methyl donor,^128^ and the enzyme has a broad substrate range for catechols including 3,4‐dihydroxybenzaldehyde (**70**), the precursor of vanillin (**71**) (Scheme 29). The codon‐optimized gene coding for human COMT was co‐expressed in two yeast strains (*S. pombe* and *S. cerevisiae*) along with microbial dehydroshikimate dehydratase and carboxylic acid reductase with the goal to generate an organism capable of de novo synthesis of **71** from glucose.^129^ Strains expressing human COMT proved to be superior for **71** production to those expressing 5 plant homologues. In flask culture, titers of **71** varied between 21 and 31 mg L^−1^ and from 12 liters of culture approximately 200 mg of pure **71** was isolated. In a subsequent study, introduction of an appropriate UDP‐glucosyltransferase into the *S. cerevisae* strain was employed to generate vanillin β‐d‐glucoside, thus avoiding vanillin toxicity. In combination with metabolic engineering approaches to maximize yields, this approach allowed vanillin glucoside titers of up to 500 mg L^−1^ to be achieved.^130^ A process based on the work described has been commercially promoted by Evolva AG (Allschwil, Switzerland) in partnership with International Flavors and Fragrances (New York, USA).

### 8.2 N‐ and S‐Methylation

**Scheme 30:**
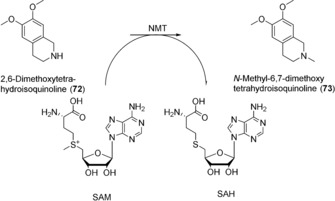
Methylation reaction catalyzed by N‐methyltransferase enzymes.

N‐Methyltransferases (NMTs) catalyze the methylation of amines at the expense of SAM.^131^ Microbial and plant enzymes have been employed for N‐methylation of small molecules, for example the preparation of *N*‐methyl‐6,7‐dimethoxytetrahydroisoquinoline (**73**; Scheme 30).^132^ Human SAM‐dependent amino acid methyltransferases have found some application in biomolecular labelling studies, since their coenzyme promiscuity allows the introduction of a non‐methyl functional group from a SAM analogue,^133^ however, as yet no human NMTs have been applied in organic synthesis.

S‐Methylation of thiopurine drugs is catalyzed by thiopurine methyltransferase (TPMT), also a SAM‐dependent enzyme.^134^ Despite its role in drug metabolism, the endogenous substrates are completely unknown. In a similar fashion to the methyltransferases, recombinant TPMT has also been exploited for the transfer of a keto group from a non‐natural SAM analogue.^135^ This will potentially allow identification of TMPT substrates.

### 8.3 O and N‐Acetylation

**Scheme 31:**
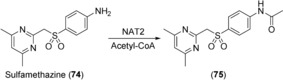
NAT2‐catalyzed N‐acetylation of sulfamethazine (**74**).

N‐Acetylation is an important pathway in the metabolism of aromatic amines, including drugs and carcinogens. An acetyl‐group is transferred from acetyl‐CoenyzmeA to the amine acceptor by N‐acetyltransferases (NATs).^136^ The two human enzymes, NAT1 and NAT2, are capable of N‐acetylation, as in the case of sulfamethazine (**74**; Scheme 31), and O‐acetylation.^137^ Procarcinogenic compounds can undergo activation catalyzed by CYPs and NATs.^138^ As yet, human NATs have not been employed for the generation of drug metabolites, however their role in the metabolic activation of promutagens makes them potentially useful in toxicological investigations.

### 8.4 Glutathione Conjugation

**Scheme 32:**
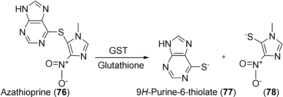
GST‐catalyzed thiolysis of azathioprine (**76**).

Glutathione‐S‐transferases (GST) catalyze nucleophilic attack of glutathione (GSH) on electrophilic substrates including drug molecules, thereby reducing their potential toxicity.^139^ Although enzymatic synthesis of GSH conjugates could, in theory, be carried out using human GSTs, there are currently no examples reported. This could be due to the availability of facile synthetic methods.^140^ In addition, GSH conjugation tends to occur spontaneously and this has been exploited in the identification of short‐lived reactive metabolites by GSH trapping.^141^ There may be potential to exploit the promiscuous thiolysis activity of GSTs in the activation of prodrugs as reported for azathioprine (**76**; Scheme 32).^142^ The authors generated a small mutant library of human GST A2‐2 based on residues thought to be important in stabilizing the transition complex and reported up to 70‐fold increase in thiolysis efficiency relative to the parental enzyme. Whether these promising results can be translated into a clinical therapeutic setting remains to be seen.

### 8.5 Sulfatation

**Scheme 33:**
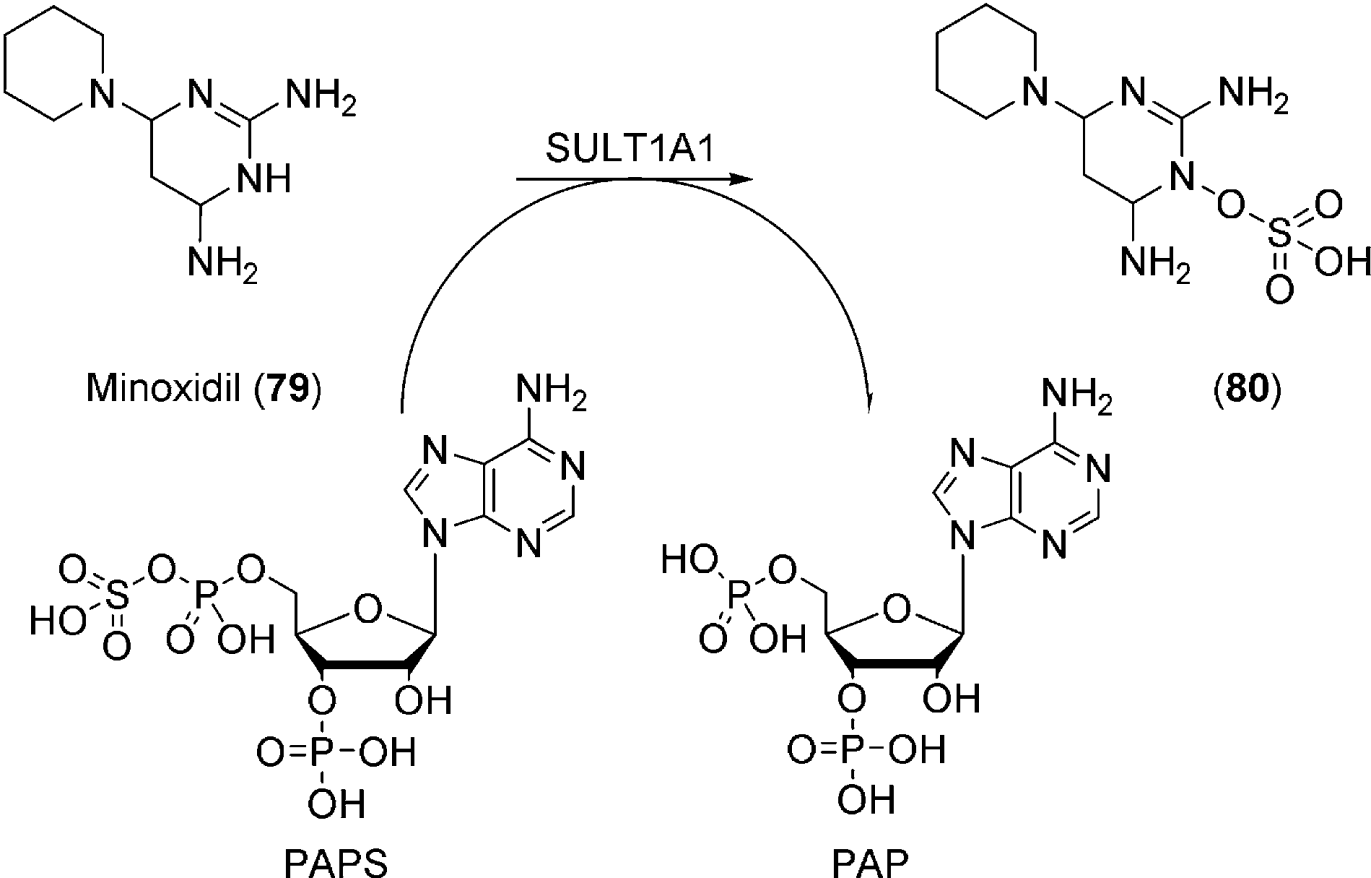
SULT1A1‐catalyzed sulfatation of minoxidil (**79**).

The addition of sulfate to alcohols or amines catalyzed by sulfotransferases (SULTs) is ubiquitous in nature, and acceptor substrates include a wide range of xenobiotics, including drug molecules and endogenous compounds such as steroids and carbohydrates.^143^ The majority of SULTs use the activated sulfate donor 3′‐phosphoadenosine‐5′‐phosphosulfate (PAPS) as cofactor as shown in Scheme 33 for the sulfatation of minoxidil (**79**) by SULT1A1.^144^ The human enzymes are readily expressed in *E. coli*.^145^ Only recently, human SULT isoforms have been expressed in *S. cerevisiae* and resting cells were applied for the preparation of sulfo‐conjugates of 7‐hydroxycoumarine, 1‐hydroxypyrene, minoxidil (**79**) and testosterone (**7**).^146^ Chemical methods for the synthesis of sulfates are readily available, although these can be challenging to implement, particularly when protection/de‐protection or sulfatation of multiple functional groups is required.^147^ Non‐human mammalian SULTs have been employed in the synthesis of heparin sulfates^148^ and rat liver AST1 in the re‐generation of PAPS for enzymatic sulfatation.^149^ It therefore appears likely that preparative examples using human SULTs will appear at some point in the future.

## 9 Ethical Aspects

In previous years the study and application of human enzymes and genes raised ethical questions that could potentially limit the application of human enzymes for research and manufacturing. Today, recombinant human enzymes are commercially available and have been used by industrial and academic laboratories for at least 15 years. Usually, their expression is based on synthetic genes derived from public sequence database information. In these cases, applied enzymes or genes are not derived from human tissue, either from specific patients who can be tracked or from anonymized individuals. It can be assumed that these genetic data, deposited in database systems at NCBI, EMBL, or in Asia had been generated from samples where consent had been provided by the individuals to generate health‐related genetic data. In contrast to commercial enzyme preparations from human tissues, where samples are standardized by mixing preparations from several different individuals (following the directive 95/46/EC of the European parliament and the council of October 24, 1995, stating that protection of individuals in respect to the processing of data and the free movement of data shall not apply to data rendered anonymous in such a way that the data subject is no longer identifiable), recombinant expression offers new opportunities to study the effects of individual single mutations and allelic variation of human genes. Therefore, newly generated pharmacokinetic data in combination with new diagnostic tests or cheap sequencing technologies might lead to sensitive health‐related genetic data that could be linked with individuals or ethnic groups in the near future. Although this might be less relevant for applications of human enzymes in organic synthesis, awareness about possible ethical conflicts should be maintained.

## 10 Summary and Outlook

Human enzymes catalyze a plethora of valuable chemical reactions as outlined in the previous chapters. Although for many of these enzymes, applications on a preparative scale have been described, their full potential has certainly not been exploited yet. So far, microbial enzymes have often been preferred since they often tend to be faster, more stable, and easier to express and, thus, were generally considered to be easier to work with. However, this is not necessarily true for the simpler biocatalysts such as ketoreductases and hydrolases, and recombinant expression provides similar or better access to human enzymes as for animal enzymes previously isolated from animal tissues and waste from meat production. Especially for the generation of authentic drug metabolites, the human enzymes constitute valuable and often unique tools. Efforts to generate humanized bacterial analogues have been only partially successful.^150^, ^151^, ^152^, ^153^ Most bacterial enzymes cover a smaller substrate range compared to only a handful of human enzymes with exceptionally broad substrate tolerance. The product range of human enzymes is also often distinct to that of bacterial homologues, which tend to produce mixtures of different metabolites. In addition, new types of reactions have been found to be catalyzed by human enzymes, which might become interesting for synthetic applications in future.^154^, ^155^ With the advances made in the field of recombinant expression technologies, current limitations in making complex human enzymes as well as access to unlimited amounts of simpler human enzymes can be addressed—a necessary step to transform them into easy to use synthetic tools. For selected high‐value applications, the relative complexity of some applied human enzymes is a fair price to be paid for their unprecedented selectivity characteristics.

## Conflict of interest

The authors declare no conflict of interest.

## Biographical Information

*Dr. Margit Winkler is Elise‐Richter fellow at Graz University of Technology and Senior Researcher at the Austrian Center of Industrial Biotechnology. She studied Technical Chemistry and completed her PhD in Organic Chemistry at the TU Graz under the supervision of Prof. N. Klempier and Prof. H. Hönig. As Erwin‐Schrödinger fellow, she joined D. O'Hagan at the University of St. Andrews. Her interests are finding, using, and improving enzymes for the synthesis of APIs or building blocks thereof*.

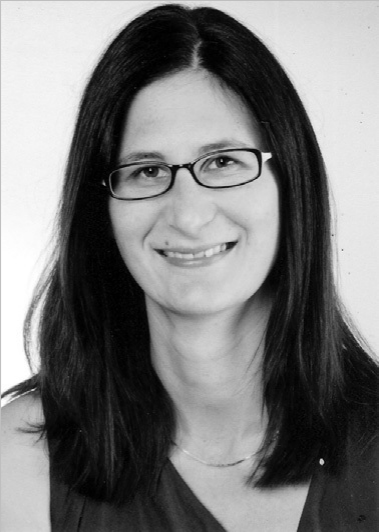

## Biographical Information

*Dr. Martina Geier is Scientist at the Austrian Centre of Industrial Biotechnology. She studied Technical Chemistry at the TU Graz. At the same university, Martina received her PhD in the group of Prof. A. Glieder for engineering human cytochrome P450 enzymes for biocatalytic applications. Her current research focuses on the generation of engineered yeasts for novel synthetic applications by implementing multienzyme pathways*.

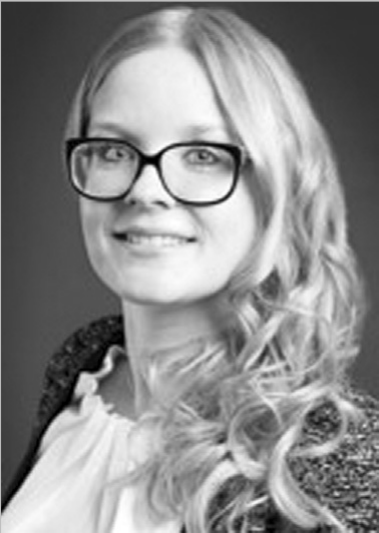

## Biographical Information

*Dr Steven Hanlon is Senior Scientist in the Biocatalysis Group at F. Hoffmann‐La Roche Ltd in Basel, Switzerland. He studied Biology before completing a PhD focused on molybdenum‐containing bacterial oxidoreductases at the University of East Anglia under the supervision of Dr. A. G McEwan. He heads a group responsible for preparing drug metabolites and chiral intermediates using whole‐cell biocatalysts with particular emphasis on oxidations*.

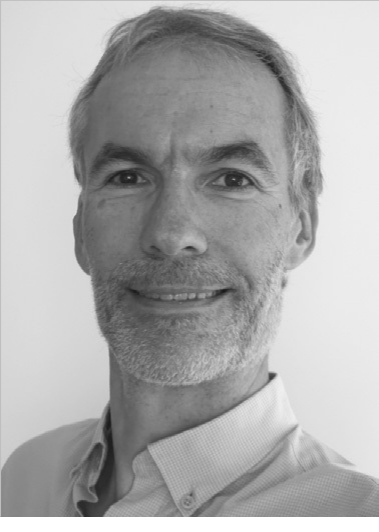

## Biographical Information

*Bernd Nidetzky is Full Professor of Biotechnology at Graz University of Technology and Scientific Director of the Austrian Centre of Industrial Biotechnology. He studied Technical Chemistry and received a PhD in Biotechnology from TU Graz in the group of Prof. W. Steiner. After a faculty position at the University of Natural Resources and Life Sciences in Vienna, he joined TU Graz in 2002. His research focuses on the mechanism and synthetic uses of different carbohydrate‐active enzymes*.

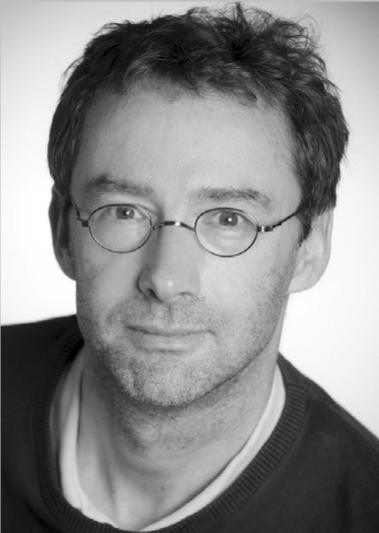

## Biographical Information

*Prof. Anton Glieder studied Biochemistry at the University Vienna and obtained a PhD in Microbiology at the University Graz under the supervision of Prof. G. Högenauer. After leading a product development department in the fruit processing industry for three years and a postdoc training at Caltech with Prof. F. H. Arnold, he obtained his habilitation in Biotechnology at TU Graz. Anton Glieder is also Cofounder of the acib GmbH and his research is focused on recombinant expression systems for eukaryotic proteins and the engineering of industrial biocatalysts and synthetic pathways*.

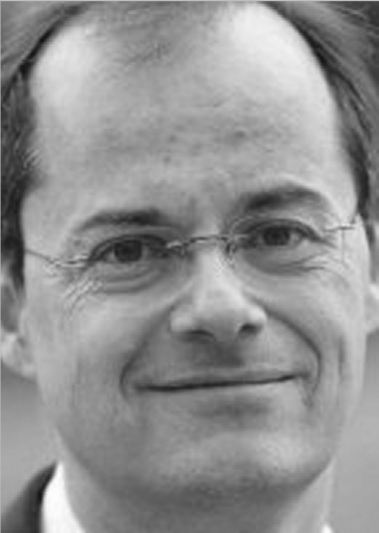

## Supporting information

As a service to our authors and readers, this journal provides supporting information supplied by the authors. Such materials are peer reviewed and may be re‐organized for online delivery, but are not copy‐edited or typeset. Technical support issues arising from supporting information (other than missing files) should be addressed to the authors.

Supplementary
