# Supplementary material for: Human Enzymes for Organic Synthesis
Source: Angew Chem Int Ed Engl. 2018 Sep 11;57(41):13406–23. doi: 10.1002/anie.201800678 (PMC6334177; doi:10.1002/anie.201800678)
Supplement: Supplementary file 1 — Supplementary [file ANIE-57-13406-s001.pdf]

Author Contributions

M.W. Conceptualization: Equal; Writing—original draft: Lead; Writing – review & editing: Lead
M.G. Writing—original draft: Supporting; Writing—review & editing: Supporting
S.H. Writing—original draft: Supporting; Writing—review & editing: Supporting
B.N. Writing—original draft: Supporting
A.G. Conceptualization: Equal; Writing—original draft: Supporting; Writing—review & editing: Supporting.
